# Self-powered multifunctional sensing based on super-elastic fibers by soluble-core thermal drawing

**DOI:** 10.1038/s41467-021-21729-9

**Published:** 2021-03-03

**Authors:** Mengxiao Chen, Zhe Wang, Qichong Zhang, Zhixun Wang, Wei Liu, Ming Chen, Lei Wei

**Affiliations:** 1 School of Electrical and Electronic Engineering, Nanyang Technological University, Singapore, 639798 Singapore; 2 CINTRA CNRS/NTU/THALES, UMI3288, Research Techno Plaza, 50 Nanyang Drive, Singapore, 639798 Singapore; 3 Center for Information Photonics and Energy Materials, Shenzhen Institutes of Advanced Technology, Chinese Academy of Sciences, Shenzhen, 518055 China

**Keywords:** Electrical and electronic engineering, Electronic devices, Organic molecules in materials science

## Abstract

The well-developed preform-to-fiber thermal drawing technique owns the benefit to maintain the cross-section architecture and obtain an individual micro-scale strand of fiber with the extended length up to thousand meters. In this work, we propose and demonstrate a two-step soluble-core fabrication method by combining such an inherently scalable manufacturing method with simple post-draw processing to explore the low viscosity polymer fibers and the potential of soft fiber electronics. As a result, an ultra-stretchable conductive fiber is achieved, which maintains excellent conductivity even under 1900% strain or 1.5 kg load/impact freefalling from 0.8-m height. Moreover, by combining with triboelectric nanogenerator technique, this fiber acts as a self-powered self-adapting multi-dimensional sensor attached on sports gears to monitor sports performance while bearing sudden impacts. Next, owing to its remarkable waterproof and easy packaging properties, this fiber detector can sense different ion movements in various solutions, revealing the promising applications for large-area undersea detection.

## Abstract (web summary)

Though thermal drawing methods are attractive for fabricating fiber-based sensor devices, existing methods allow limited access to low viscosity and low modulus materials. Here, the authors demonstrate a two-step soluble-core fiber fabrication method with wide applicability to soft polymer materials.

## Introduction

Fiber drawing from a preform has traditionally been exploited to produce the extended lengths of optical fibers as telecommunication waveguides^1,2^ to deliver enormous amounts of light data over the kilometer length scale^3–5^. Though initially developed for optical fibers, preform-to-fiber thermal drawing technique is an inherently scalable manufacturing method that produces functional fibers with the integration of a broad range of materials^6–8^, including glasses, semiconductors, metals, and polymers. It has enabled a wealth of functionalities, including material engineering^9–11^, optical sensing^12,13^, neural sensing^14,15^, thermal detection^16,17^, chemical sensing^18,19^, acoustic emission^20,21^, and many more^22^. Prospectively, such functional fibers can be a promising candidate for soft electronics^23–27^ with the consideration of their unique advantages of high dynamic bending elasticity^28^, stretchability^29^, and high mechanic strength^30,31^. Despite the myriad of available working principles which the soft electronics are mainly developed from such as piezoresistive effect^32,33^, capacitance variation effect^34^, piezoelectric effect^35^ etc., an emerging technique triboelectric nanogenerator (TENG)^36^ utilizing the triboelectrification effect has enabled wide spread applications in self-powered sensors^37,38^. It detects mechanical stimulations from both static and dynamic processes through the voltage and current output signals of the TENG, converting ambient mechanical energy into electric energy via a coupling effect of contact electrification and electrostatic induction^39,40^. Among the demonstrated device form factors, fiber-shaped TENGs are able to fully utilize fiber electronics’ advances of large specific surface area while being lightweight, as well as its feasibility to achieve scalable fabrication^41^. The main requirement for co-drawn material selection is that the materials should exhibit compatible viscosities at the drawing temperature^42^. However, the use of soft materials such as elastomers is not compatible with the thermal drawing process, and the fiber geometry is difficult to maintain, due to the low modulus, low viscosity, and high adhesiveness of elastomers under high temperature^30^.

Thus, with the effort in this work, a universal two-step soluble-core fiber fabrication method has been successfully developed that is applicable to a wide range of soft materials to be compatible with the preform-to-fiber thermal drawing process while maintaining fiber structures and interfaces. This approach breaks the constrain in polymer material selection for the conventional thermal drawing technique, further extending to low viscosity rage, thus may enable a broad range of low viscosity functional materials (e.g., synthesized self-healing gels) to be developed into fiber devices. The first step is the co-drawing of styrene-ethylene-butylene-styrene (SEBS, a typical elastomer) shell and polyvinyl alcohol (PVA) core into a thin fiber that defines the initial structure. The second step dissolves the PVA core to obtain a hollow SEBS tubular fiber. Furthermore, by infiltrating liquid metal into the hollow SEBS fibers, TENG fibers are thus fabricated. This ultra-stretchable conductive fiber offers an outstanding stretchability which is still conductive at 1900% strain and can bear 1.5 kg load/impact freefalling from 0.8-m height. Moreover, working as soft electronics, it is self-adapting on regular and irregular surfaces, which is ideally suitable for monitoring sports performance and training while bearing strong and sudden impacts when attached on sports gears. For the proof of concept, we construct both a 2D fiber net on a baseball glove to locate the hitting points with different catching speeds and a 3D fiber net on a football to perform the sensing function of a spherical coordinate. More importantly, different from the previously reported TENG fibers which were fabricated through dip coating^43,44^, spray coating or deposition^45^ of functional layers onto textiles fibers^46,47^, and wet- or electro-spinning^48^, the fabricated TENG fibers in this work perform a better stability, easy packaging, and waterproof, leading to the realization of an ion movement detector in different solutions, potentially for large-scale underwater applications.

## Results

### Soluble-core fabrication of super-elastic fibers

**Fig. 1:**
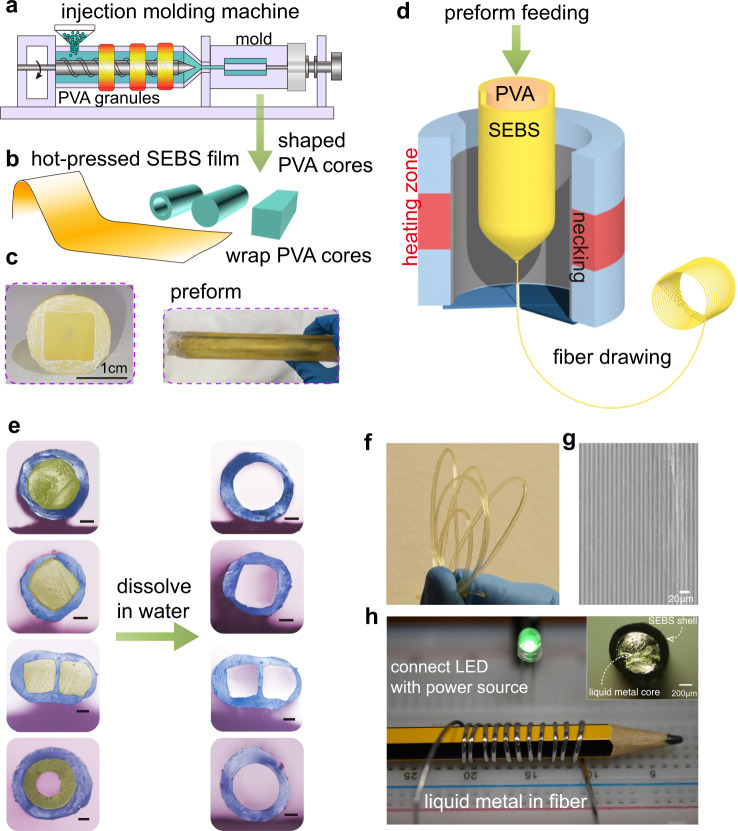
Soluble-core fabrication of the super-elastic conductive fiber through thermal drawing method. **a** PVA pellets were reshaped into rods with different shapes by injection molding machine. **b** To wrap shaped PVA cores with SEBS films. **c** The prepared preforms after consolidation. **d** Schematic of the fiber drawing process in the thermal fiber drawing tower. Preforms with different shaped cores were pre-fabricated, and the all-solid preform was heated in the furnace till soft and then drawn into fibers. **e** Cross-section photos of different shaped PVA cores before and after dissolving the inner cores in water. The inserted scale bars are 200 μm. **f** Optical image of the resulting fibers. **g** The microstructure fabricated on fiber surface with the scale bar of 20 μm. **h** The formation of ultra-flexible conductive fiber. By injecting liquid metal GaIn eutectic alloy into the hollow channel and connecting copper wires on both ends of the fiber as electrodes, an ultra-flexible and ultra-stretchable conductive fiber was formed. A green LED lighted up when connecting to the power source via a piece of conductive fiber that was coiled on a pencil. The inset is the cross-section view of the liquid metal core fiber with the scale bar of 200 μm.

The fabrication approach to produce these fibers is illustrated in Fig. 1. To form the PVA core-SEBS shell preform, SEBS pellets were hot-pressed into thin films, while PVA pellets were reshaped into rods with different shapes (e.g., cylinder, cuboid, tube etc.) by injection molding machine as shown in Fig. 1a. The PVA rod core was wrapped tightly by the SEBS films (Fig. 1b). Then the preform was consolidated in vacuum oven to remove moisture and air gaps, and the well prepared preforms are shown in Fig. 1c. The resulting all-solid preform was heated in a two-zone furnace till soft and then drawn into fibers with precisely controlled diameters from micrometer to millimeter, as shown in Fig. 1d. For the convenience of subsequent fabrication steps, we kept the outer diameter to be ~0.8 mm. After collecting the fibers, they were cut into segments, and immersed in 80 ^o^C hot water to fully dissolve the inner cores. Figure 1e shows the cross-section photos of different shaped PVA cores before and after dissolving the inner cores in water. The dissolving rate is about 10 μm/min in room temperature. To reduce the dissolving time, an effective approach is to fabricate tubular-shaped PVA core (bottom row in Fig. 1e), compared to the all-solid PVA cores. The dissolving parameter data sheets are listed in Supplementary Table 1 and Supplementary Table 2. As shown in Fig. 1f, the resulting fiber is very flexible with a continuous hollow channel running within the entire fiber length. Microstructures were hot-pressed onto the fiber surface to increase the surface area as shown in Fig. 1g. Then, the liquid metal GaIn eutectic alloy was injected into the hollow channel, followed by the connection with copper wires on both fiber ends, forming an ultra-flexible and ultra-stretchable conductive fiber. Figure 1h exhibits the outstanding flexibility of the formed conductive fiber in which such a conductive fiber was coiled on a pencil, while maintaining the electric connection to light up a green LED. The insert is the cross-section view of the liquid metal core fiber with the inner diameter of 600 μm. This technique is not only applicable to the incorporation of liquid metal into fibers, but also extended to other electronic devices that can be developed in a similar fashion, such as ionic liquid and other functional solutions. Also, increasing the number of channels within a single fiber will potentially lead to even higher composite electrical applications.

### Super-elastic conductive fibers working as TENGs

**Fig. 2:**
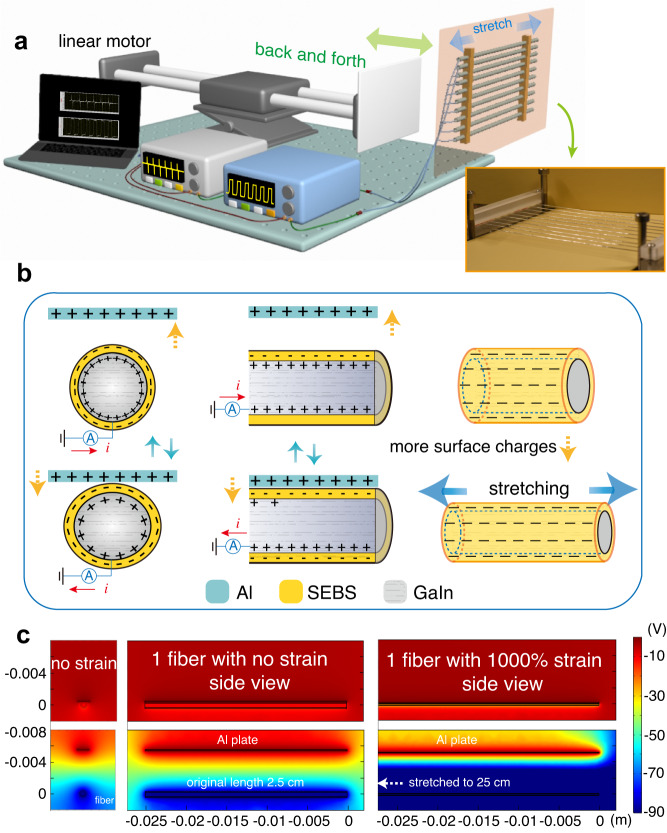
Super-elastic conductive fibers working as TENG to harvest mechanical energy. **a** Schematic of the test platform. The fibers were fixed parallelly by a pair of clamps on a vertical optical board. The strain was manipulated by adjusting the distance between the clamps. The aluminum plate contacted the fibers back and forth controlled by the linear motor. **b** Working mechanism of the ultra-stretchable fiber TENG. When the aluminum plate gets close to and contacts the fiber surface, the positive charges on the liquid metal core at the interface between SEBS shell decreases to keep the electrostatic balance. When the aluminum plate moves away from the fiber surface, the positive charges on the liquid metal core surface increase. When fibers are stretched, the surface area increases, the charges on the surfaces increase, and the electrical output increases. **c** Simulation of one fiber working condition under no strain and 1000% strain.

To quantitively study the electrical output of the ultra-stretchable conductive fiber device working as TENG, we constructed a test platform which could both perform a cycling contact-separate working mode and adjust the fiber strains. Schematic of the test platform is shown in Fig. 2a. The fibers were fixed parallelly by a pair of clamps on a vertical optical board. The strain was manipulated by adjusting the distance between the clamps, and screws fixed on the breadboard were utilized to hold the clamps from moving/displacing. The aluminum plate contacted the fibers back and forth controlled by the linear motor, and the traveling speed was set as 0.1 m/s. Electrometers were connected with copper wires from the conductive fibers to collect the corresponding electrical output. The working mechanism of the ultra-stretchable fiber-based TENG under this test mode is shown in Fig. 2b. When the aluminum plate gets close to and contacts the fiber surface, the positive charges on the liquid metal core at the interface between SEBS shell decreases to keep the electrostatic balance. The direction of the current is “+”. When the aluminum plate moves away from the fiber surface, the positive charges on the liquid metal core surface increases. The direction of the current changes to “-”. Full working cycle mechanism schematic with corresponding signal indications is shown in Supplementary Fig. 4. Furthermore, under an ideal condition, the surface charges and surface area are in a radicand relation with the fiber length (detailed discussion presented in Supplementary Discussion 1), so when fibers are stretched, the surface area increases, charges on the surfaces increase and the electrical output increases. Other factors (e.g., fiber thickness and humidity) can also contribute to the charge density^49^, but have negligible influence on our fiber condition, so we simply discuss in Supplementary Discussion 1. Simulation results of one fiber working under no strain and 1000% strain are shown in Fig. 2c. Charge density was extracted from experimental results as 0.334 μC/m^2^, and 2.44 μC/m^2^, respectively. Obviously, the electrical output under the stretching condition is larger than that with no strain. More simulation results are shown in Supplementary Figs. 6 and7.

### Characterization of super-elastic conductive fiber-based TENG

**Fig. 3:**
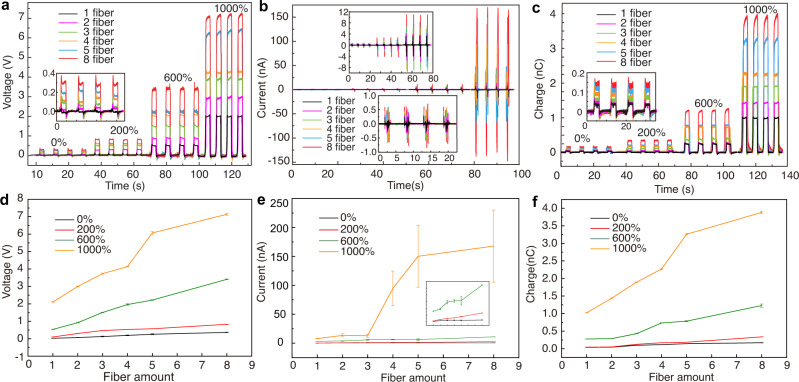
Electrical output of different numbers of fibers working as TENGs under different strains. **a** Open-circuit voltages of fibers under strains 0, 200, 600, and 1000%. The output voltage increases with the number of fibers and the applied strains. Inset: the enlarged figure of open-circuit voltages under 0% strain. **b** Short-circuit currents of fibers under strains 0, 200, 600, and 1000%. The output current increases with the number of fibers and the applied strains. Inset: the upper one is the enlarged figure of short-circuit currents under 0, 200, and 600% strains, and the bottom one is the enlarged figure under 0% strain. **c** The transferred charge quantities of fibers under strains 0, 200, 600, and 1000%. The quantity of charges increases with the number of fibers and the applied strains. Inset: the enlarged figure of transferred charges under 0% strain. The motor speed was set as 0.1 m/s when tested. **d**–**f** Extracted peak values with error bars of voltage, current, and transfer charge of different numbers of fibers under different strains from **a**–**c**, respectively. Error bars represent standard deviation, *n* = 8 independent replicates.

Electrical output of different numbers of fibers working as TENGs under different strains were measured and shown in Fig. 3. The test platform is illustrated in Fig. 2a, and the motor speed was set as 0.1 m/s. Figure 3a plots the open-circuit voltage outputs of fibers under strains of 0, 200, 600, and 1000%, with the number of fibers to be 1, 2, 3, 4, 5, and 8, respectively. Starting from the original fiber length of 25 mm, the output voltage increases with the number of fibers and the applied strains. Eventually, eight fibers with 1000% strain can provide an output of ~7.5 V. Figure 3b, c plot the short-circuit currents and the transferred charge quantities of fibers under strains of 0, 200, 600, and 1000%, with the number of fibers to be 1, 2, 3, 4, 5, and 8, respectively. Both the output current and the quantity of charges increase with the number of fibers and the applied strains. In order to see the trend more clearly, in Fig. 3d–f, we extracted the peak values of voltages, currents, and transfer charge quantities of different numbers of fibers under different strains from Fig. 3a–c, respectively. It can be observed that the electrical outputs increase with the number of fibers under all strain conditions and are in a positive correlation with the applied strains. Stretchable fibers gripped by clamps work as a fix-fix string vibration model, so the vibration frequency increases with its strain. It is indicated by the statistical analysis that error bars become larger with increase of the applied strains and the number of fibers, especially in current signals which are obtained through the rate at which charge flows through a given surface. This random vibration cannot be avoided in the clamp-fiber-clamp system. Moreover, because of the superposition of randomness, it becomes more obvious when there are more fibers. Detailed explanation is provided in Supplementary Discussion 2.

**Fig. 4:**
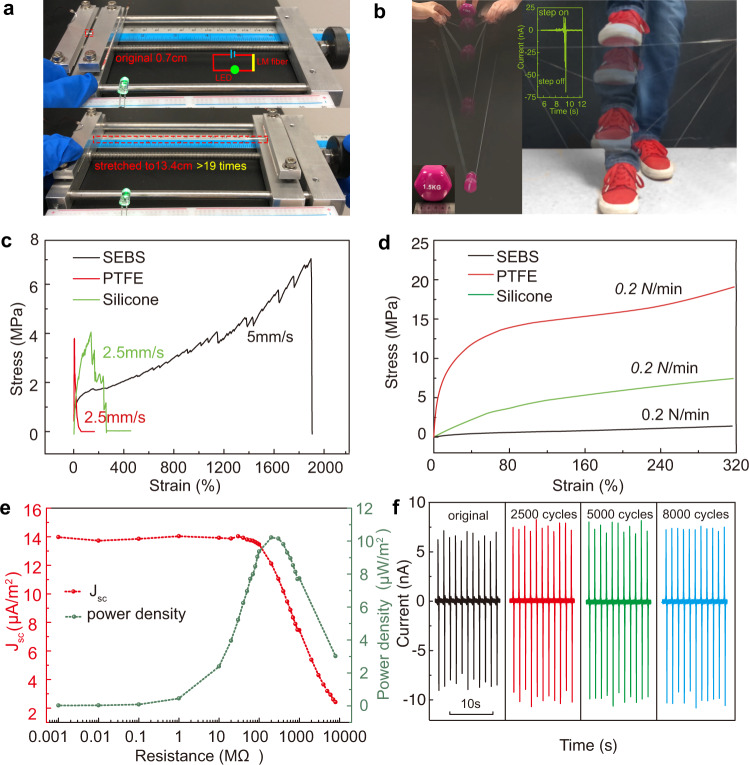
Stretchability and durability of the super-elastic conductive fibers. **a** The liquid metal fiber is still conductive when stretched to up to 19 times. See Supplementary Movie 1. **b** Left: 10-cm liquid metal fibers bear the dynamic impact caused by a 1.5 kg load free-falling motion from 0.8-m height. See Supplementary Movie 2. Right: The resulting fibers can bear the impact and deformation caused by human activities such as stepping. Inset: the collected current signal of foot stepping (~75 nA). **c** Tensile test of SEBS, PTFE, and silicone hollow fibers. SEBS hollow fiber shows the best stretchability. **d** DMA test of SEBS, silicone, and PTFE hollow fibers to obtain the Youngs’ modulus of 1.6, 5.8, and 204.1MPa, respectively. **e** Power density with different out loads. Corresponding measured voltage is plotted in Supplementary Fig. 10. **f** Current output with 2500, 5000, and 8000 contact-separation cycles under 0.3 m/s speed to show the durability of the super-elastic conductive fibers.

Though in the electrical output characterization process the strain was only set up to 1000% to obtain a stress uniformity in a moderate scope, the liquid metal fiber was still conductive when stretched to up to 19 times (Fig. 4a and Supplementary Movie 1). Because of the super-elastic nature, it can also bear the dynamic and sudden impact caused by a 1.5 kg load that is free-falling from 0.8-m height, as shown in Fig. 4b and Supplementary Movie 1with a group of six 10-cm-conductive fibers. Moreover, to reveal the potential of using such fiber to perform as human activity sensor and mechanical energy harvester, it can also bear the impact and deformation caused by human activities such as stepping, as demonstrated in Fig. 4b. The inset of Fig. 4b shows the collected corresponding current signal of foot stepping (~75 nA). To quantitatively study the stretchability, strain-stress tests of SEBS, PTFE, and silicone hollow fibers were carried out by both mechanical testing machine (MTS) and dynamic mechanical analysis (DMA). As summarized in Fig. 4c, SEBS hollow fiber shows the best stretchability performance, which reaches 1900% strain. As the working distance in DMA machine was not long enough for the breaking test, strain-stress curves in Fig. 4d were used to obtain the Youngs’ modulus 1.6, 5.8, and 204.1 MPa for SEBS, silicone, and PTFE hollow fibers, respectively. Figure 4e is the output power density of a six 10-cm-conductive fiber-based TENG with different external loads under 0.1 m/s contact-separate working speed. The output current decreases with the increasing of external resistances, while the voltage follows a reverse trend (Supplementary Fig. 10), which is due to the Ohm’s law. Thus, the instantaneous power density increases firstly and then drops, being maximized at a load resistance of ∼200 MΩ, corresponding to a peak power density of ∼10.2 μW/m^2^. Durability is one of the key factors to ensure the long-term stability of devices, and Fig. 4f plots the current output with 2500, 5000, and 8000 contact-separation cycles under 0.3 m/s. The output keeps at around +7 nA to −10 nA, indicating a stable operation in the testing period, and the full data is shown in Supplementary Fig. 11. More durability tests of fibers with 200% strain and 1000% strain are shown in Supplementary Fig. 12 and Supplementary Fig. 13.

### Conformal fiber net for monitoring sports performance and training

**Fig. 5:**
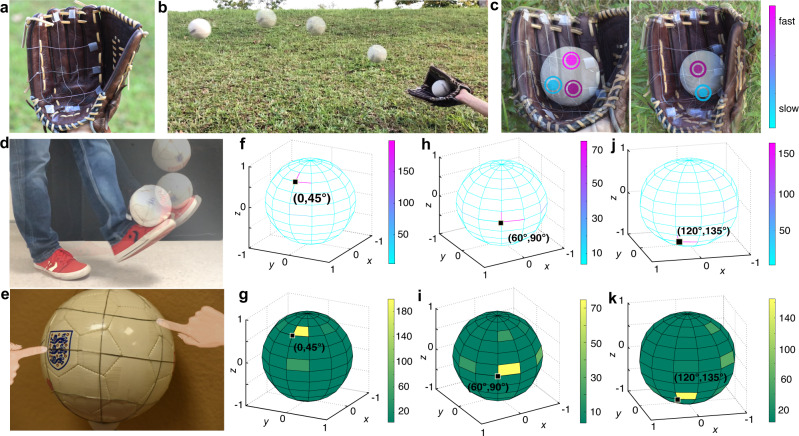
Applications as an adaptive sensor net on regular and irregular shaped surfaces for monitoring sports performance and training. **a** A fiber-based sensing net that was conformally adapted on the 2D inner surface of a baseball glove to locate the hitting points with different catching speeds. **b** Photo to show the ball-catching using functional baseball glove. **c** The detection to locate the hitting points with different catching speeds when the baseball was caught by the glove. Colors indicate different catching speeds. **d** Conductive fibers on a 3D surface of a football to perform the sensing function of a spherical coordinate. **e** Schematic to show the cross point when pressed by fingers. **f**–**k** When a cross point was pressed, the corresponding signal was visualized on the spherical coordinates. **f** and **g** Point (0°, 45°) was pressed. **h** and **i** Point (60°, 90°) was pressed. **j** and **k** Point (120°, 135°) was pressed. The color indicates the signal value, and if both coordinate values are high enough, the upright coordinate line segments or area would be highlighted, which means that this cross point is pressed.

Based on the advances of its ultra-flexibility and ultra-stretchability, the fabricated conductive fiber offers an outstanding conformability and can be adapted to regular and irregular shaped surfaces. In particular, the nature of monitoring sports performance and training requires the direct contact with the training facilities,^50^ while maintaining stable sensing performance under sudden deformation and strong external impact. The fabricated conductive fiber is therefore a promising candidate to meet these strict requirements. So, we first constructed a fiber-based sensing net that was conformally adapted on the 2D inner surface of a baseball glove to locate the hitting points with different catching speeds. As demonstrated in Fig. 5a–c, when the ball was caught, hitting points were detected, and the catching speed was indicated by analyzing the current value differences, as lower catching speed results in a smaller current signal, and higher catching speed leads to a larger current output. (Color differs depending on the speed in the schematic in Fig. 5c).

The second demonstration is to apply these conductive fibers on a 3D surface, such as the surface of a football, as shown in Fig. 5d, e, and this fiber net on the football surface exactly performs the sensing function of a spherical coordinate.^51^ Fibers attached on the football surface formed both the longitude line and the latitude line, and each cross point of the fiber was regarded as a coordinate point in the spherical coordinate system. For testing convenience, we performed the finger touch test as shown in Fig. 5e. We used the current value to detect and identify the point under impact based on the longitude lines of 0° (360°), 60°, 120°, 180°, 240°, and 300°, and latitude lines of 45°, 90°, and 135°. As the fabricated conductive fibers offered an ultra-sensitivity to environment motion change, we adopted a special data processing method to avoid possible noise jamming. When a finger touched a point, current signals from all nine lines were received. For each line, we extracted the signal values of both touched and untouched states, and then took the ratio of touched value/untouched value as the line value *R*. Thus, for one certain touch, we got 9 *R* from nine fibers. Our target was to detect which cross point was touched, so we transfered line values to the cross point values by taking the product value of the two crossed lines’ *R* as the point value. For instance, the detected value for point (30°, 120°) is *R*_*(lon30°, lat120°)*_ = *R*_*lon30°*_ × *R*_*lat120°*._ So for one certain touch, we got 18 detected values for the 18 cross points, respectively. Then, to visualize the entire sensing net, we assigned these 18 values to the corresponding positions on the spherical coordinate, as shown in Fig. 5f, g with the maxmum value appeared at (0°, 45°) and the most bright area was the upright line segment and area of point (0°, 45°). Based on this sensing principle, Fig. 5h, i show the detecting results when point (60°, 90°) was under impact, while Fig. 5j, k show the detecting results when point (120°, 135°) was under impact. Movies to show the full coordinate points on the spheres can be found in Supplementary Movie 3.

### Super-elastic conductive fiber for ion movement detection in solutions

**Fig. 6:**
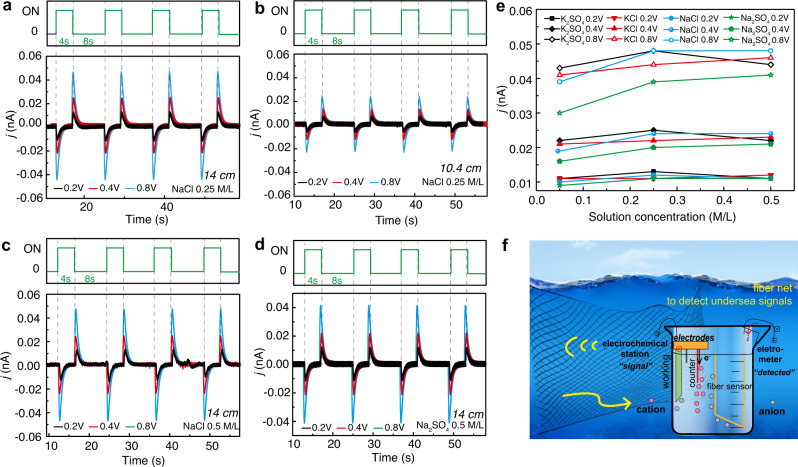
Super-elastic conductive fiber for ion movement detection in solutions. **a** A 14-cm-long fiber was immersed in 0.25 M/L NaCl solution and the solution was applied with DC rectangular wave signals of 0.2, 0.4, and 0.8 V from the electrochemical workstation. The upper green line shows the source signal on-off and the below correspondingly shows the detected signal from our functional fiber. **b** A 10.4-cm-long fiber was immersed in 0.25 M/L NaCl solution and the solution was applied with DC rectangular signals of 0.2, 0.4, and 0.8 V. **c**, **d** A 14-cm-long fiber was immersed in 0.5 M/L NaCl solution and 0.5 M/L Na_2_SO_4_ solution under 0.2, 0.4, and 0.8 V applied DC rectangular wave signals. **e** Summary of a 14-cm-long fiber detecting results in different concentration solutions and different applied DC rectangular wave voltage signals. It shows that the detecting results are majorly related to the applied signal value which represents the ion movement. **f** A schematic of the prospected application for undersea detecting with the inset schematic to explain the self-powered detecting process.

Another merit of fiber devices is that it is much easier to be packaged (only two ends) compared with film devices^52,53^ and on chip devices^54^, which is particularly suitable for underwater applications. As far as reported, TENGs working underwater mainly make use of the mechanical energy of waterwaves^55^, and a recently work has developed a bionic film device by collecting underwater mechanical energy through utilizing water flow in device channels^56^. Besides the mechanical movement induced static charge variations between/among components of TENGs, we found that ion movements and concentration changes in solutions could also result in electrical output, which is accompanying TENG waterwave mechanical energy harvesting. This phenomenon could be utilized to detect underwater electrical signals and collect related energy. Here we demonstrate a way to make use of this phenomenon based on our super-elastic conductive fibers. To build up a controllable ion movement environment in solution, we constructed a three-electrode system connected with the electrochemical workstation using a carbon cloth as working electrode, a platinum wire as the counter electrode, and Ag/AgCl as the reference electrode, as schematzed in Fig. 6f. When “+” source signal (upper figures in Fig. 6a–d) is applied, the electrochemical station gives out electrons *e*^*-*^ with negative charges through the counter electrode (Pt wire). So, cations in the solution move from other locations toward the counter electrode, and anions distribute more at other locations in the solution. As there are negative anions outside the detecting fiber, positive charges move into fiber to reach balance. Thus, current direction is into the fiber from the electrometer, which appears as negative in the detecting signals presented in the bottom part of Fig. 6a–d. When the applied source signal is fully applied, the solution system gradually reaches a balance state, and the detected peak recovers to 0. When the “+” signal is off, an opposite process happens, and the detected signals become opposite. (Detailed discussion is shown in Supplementary Fig. 16.)

Factors including source amplitude, detecting length of fibers, solute types, and solution concentrations may affect the detecting results. As revealed in Fig. 6a–d, the value of sources signals obviously has an impact on the detecting results (0.8 > 0.4 > 0.2 V). So we firstly investigate how the detected signals are influenced by the immersed fiber length. In Fig. 6a, a 14-cm-long fiber was immersed in 0.25 M/L NaCl solution, and the solution was applied with DC rectangular wave signals of 0.2, 0.4, and 0.8 V through multi-potential steps mode from the electrochemical workstation. To make a comparison, in Fig. 6b, a 10.4-cm-long fiber was immersed in the same 0.25 M/L NaCl solution and the solution was also applied with DC rectangular wave signals of 0.2, 0.4, and 0.8 V. The detected signals are smaller than those in Fig. 6a under the same applied voltage, indicating that the detected signal value is majorly related to the effective fiber length immersed in solution. Next, in Fig. 6c, d, the effect of solute difference was investigated by immersing a 14-cm-long fiber in 0.5 M/L NaCl solution and 0.5 M/L Na_2_SO_4_ solution separately, under 0.2, 0.4, and 0.8 V DC rectangular wave signals. The detecting results obviously differ with different solutes under 0.8 V, and less obviously under low source signal values. Figures 6a, c investigated the solution concentration as a factor, and the detecting results did not differ obviously with different concentrations of the same solute. Full test results were extracted and summarized in Fig. 6e, verifying the above results and conclusions. Firstly, the source signal value obviously played a more important role than other factors, as the curves clearly distributed in three groups according to signal values (0.8 > 0.4 > 0.2 V). Secondly, the curves for four solutes differed little under 0.2 and 0.4 V signals, and differed larger under 0.8 V signals, but were still insufficient to be clearly distinguished with each other. Lastly, the curves did not show an obvious positive relation between the solution concentration and the detecting results. The reasons for the unclear regularity might be due to the facts that the conductivities of solutions with different solute types are different and the ion movement conditions differ with ion types (their carried charges and the ion weights may affect their movement under electric field). Therefore, this systematic study confirms that the immersed fiber length and the source signal values are the major factors affecting the detecting results. The solute type and solution concentration are the minor factors. Figure 6f demonstrates the prospected application for large-area undersea monitoring. The super-elastic conductive fibers can detect undersea electrical signals caused by ion movements and concentration changes, while collecting the energy simultaneously. The inset is a schematic to explain the self-powered detecting process.

## Discussion

Ultra-stretchable SEBS hollow fibers were fabricated using the proposed soluble-core method. By incorporating liquid metal into the inner core, a super-elastic conductive fiber was achieved with easy packaging. Combining the effects of triboelectrification caused by surface contact and principles of electrostatic induction, the ultra-stretchable conductive TENG fiber can be attached to 2D and 3D surfaces working as a self-powered sensor while bearing strong and sudden impacts. Thanks to its intrinsic advantages of waterproof and easy packaging, the as-fabricated fiber was also utilized to study the electrical signal change and ion concentration variations in different solutions, indicating the potential as an underwater self-powered monitoring net. This newly proposed fabrication approach and the combination with TENG provide more possibilities to achieve various in-fiber structures and a viable path to introduce soft material enabled electronic devices into thermally drawn fibers, which allows more space for further large-area high-dimensional device integration. Especially, inspired by the effectivity of detecting ion variation related signals, multi-position underwater motion monitoring system can be expected.

## Methods

### Materials

SEBS pellets were G1643 from Kraton. PVA pellets were G-polymers 8049, 8042, and 8077 P from NIPPON GOHSEI which permit a temperature window (between *T*_m_ and decomposition temperature) for thermal processing. GaIn eutectic, NaCl, and KCl were from Alfa Aesar. K_2_SO_4_ and Na_2_SO_4_ were from Aladdin.

### Fabrication of PVA core-SEBS shell preforms

PVA pellets were filled in the heating barrel of the injection machine, and melted at 200–225 °C for 3–5 min. Then the melted PVA was injected to the mold under pressure of 0.7 MPa. SEBS pellets were firstly hot-pressed into thin film under 190 °C for 8 mins, then cropped to rectangular shape. SEBS films were rolled outside PVA cores tightly. And the PVA cores were shaped to cylinder, cuboid, tubular, and other shapes depending on the mold shapes. For dual core preform, the two cores were wrapped separately and then combined to form one preform. The wrapped preforms were consolidated in vacuum oven at 110 °C.

### Fiber drawing

The fibers were fabricated by the thermal drawing process by placing the preform in a two-zone heating furnace, where the top and bottom zones were heated to 150 and 350 °C, respectively. The preform was fed into the furnace at a rate of 3 mm min^−1^ and drawn at a speed of 1 m min^−1^, which resulted in a draw-down ratio of 18. Tens of meters of fibers were collected from each draw.

### Dissolving inner core and conductive fiber fabrication

The collected fibers were cut into segments and immersed in 80 ^o^C hot water for overnight to fully dissolve the inner cores. After completely drying off, we used a syringe to inject GaIn eutectic into the hollow channels. Then copper wires were inserted to both ends and glues were used to seal the connections.

### Visualization of the sensing results on the football

To visualize the entire sensing net, a spherical coordinate system framework was constructed by 12 longitude lines × 12 latitude lines, which both supported a sphere surface shape and included all 18 detect cross points. We assigned 18 R values to the corresponding positions on the spherical coordinate, and took the detected minimum value to assign the rest coordinate points on the sphere. Then the coordinate sphere was colored depending on the coordinate point values. If both coordinate values of a point are high enough, the upright coordinate line segments or area would be highlighted, which means this cross point is pressed. The reason why the supporting lines on the sphere coordinate structure are more than we tested is that the sphere surface would be tangled when the supporting longitude and latitude lines were removed. So we just kept them and assigned them the minimum value.

### Electrochemical tests

A fiber holder with some groves on the plastic plate surface (Supplementary Fig. 17) was firstly fabricated to hold the soft fiber and ajust the immersing length in solutions. The fiber was placed in the grove, and connected one end by copper wire with the working electrode from the electrochemical working station. The electrical source was applied through a three-electrode system. We used the multi-potentials test mode in the electrochemical working station to apply signals. Various concentration solutions were injected to the beaker as the electrolyte.

### Characterization and measurement

A linear motor (LinMot E1100) was used to apply a periodic contact-separation working cycle. The speed was set to be 0.1 m/s. The open-circuit voltage, short-circuit charge, and short-circuit current of the fibers were measured by a Keithley 6517B electrometer, and a Stanford 570 current preamplifier. The scanning electron microscope (SEM) images were taken by JEOL field emission SEM (7600 F). The obtained cross-section optical micrographs were taken by Olympus BX51. DMA test was carried out by TA Q800. Uniaxial tensile test was examined by MTS Criterion Model 42.

## Supplementary information

Supplementary Information

Description of Additional Supplementary Files

Supplementary Movie 1

Supplementary Movie 2

Supplementary Movie 3

## Data Availability

The data that support the findings of this study are available from the corresponding author upon reasonable request.
