# Supplementary material for: Self-powered multifunctional sensing based on super-elastic fibers by soluble-core thermal drawing
Source: Nat Commun. 2021 Mar 3;12:1416. doi: 10.1038/s41467-021-21729-9 (PMC7930051; doi:10.1038/s41467-021-21729-9)
Supplement: Supplementary file 1 — Supplementary Information [file 41467_2021_21729_MOESM1_ESM.pdf]

Supplementary Information

Self-powered multifunctional sensing based on super-elastic fibers by soluble-core thermal drawing

Mengxiao Chen†, Zhe Wang†, Qichong Zhang, Zhixun Wang, Wei Liu, Ming Chen and Lei Wei*

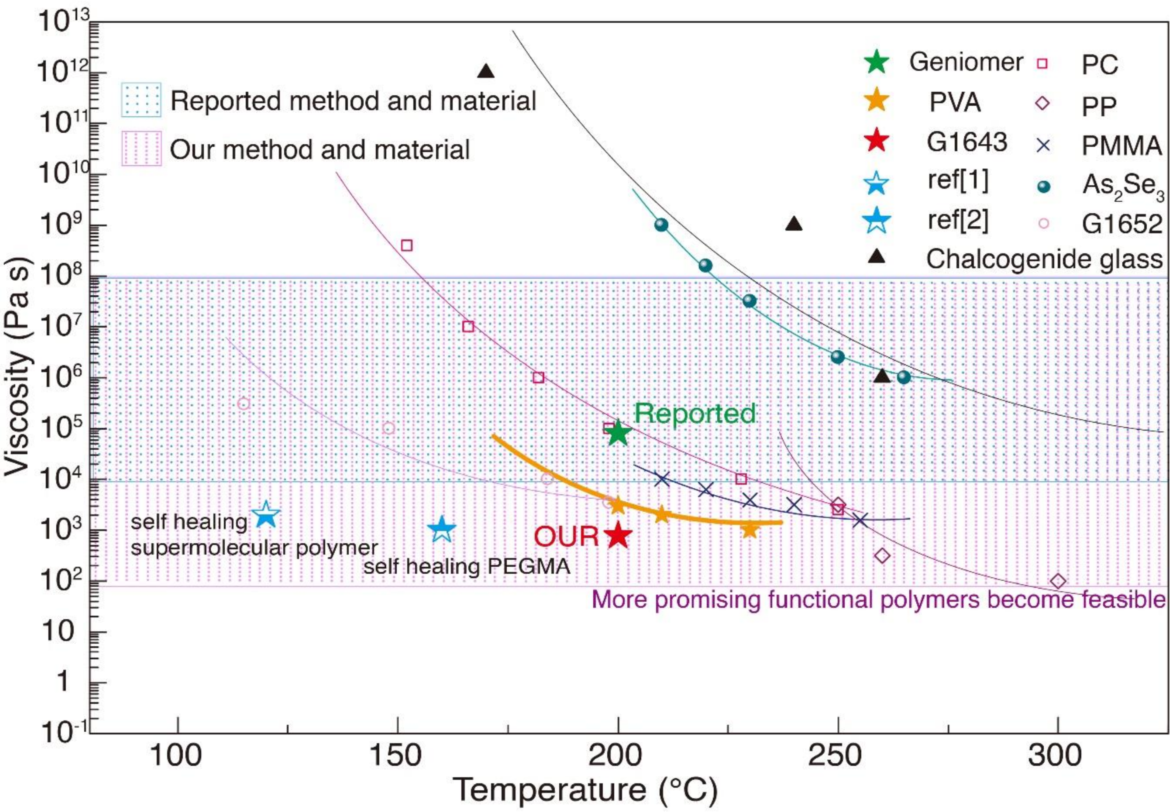

Supplementary Fig. 1 **Comparison of the accessible material ranges between the conventional thermal drawing process (blue region) and the newly developed soluble-core thermal drawing process in our work (pink region).**[1, 2]

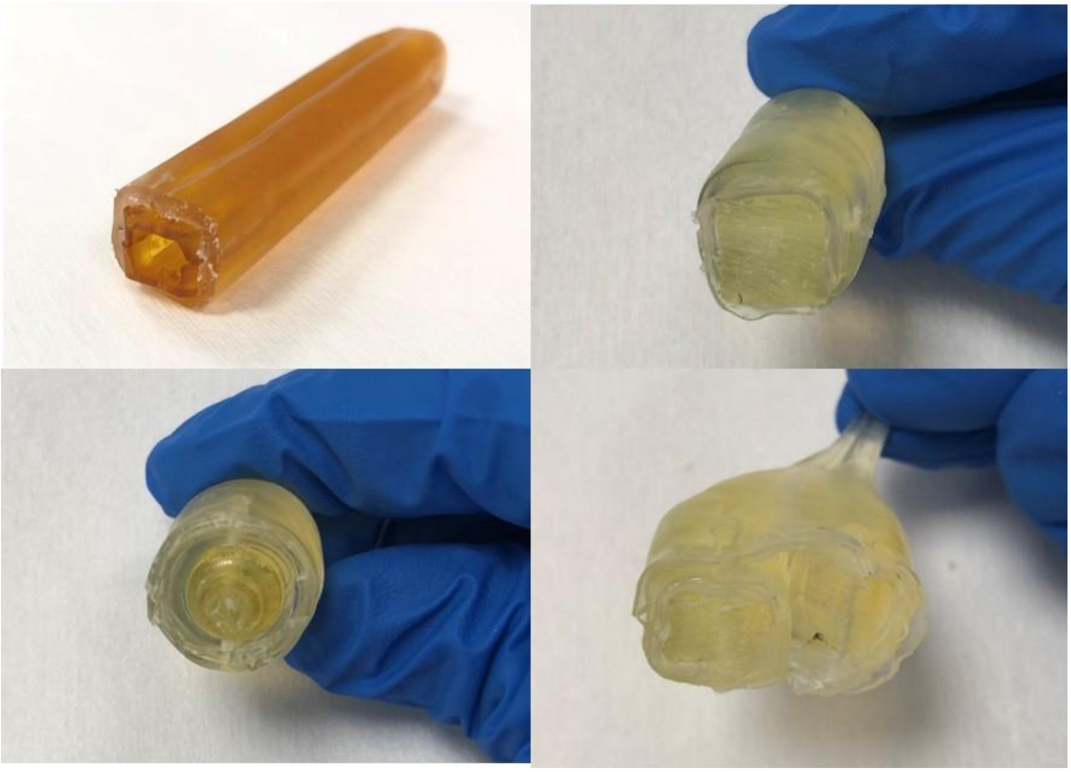

Supplementary Fig. 2 **Photos of preform and preform ends in different shapes**. The wrap and roll method is not only suitable for round shape, but also utilized to construct rectangular core and dual core.

Supplementary Table 1 **Thinner film G-polymer dissolving time in water with different temperatures.** (From NIPPON GOHSEI)

Thickness of samples	30um		100um	
Temperature (degree C)	Breaking time	Absolute dissolve time	Breaking time	Absolute dissolve time
5	16 sec.	44 sec.	2min.30sec.	3min.45sec.
20	6 sec.	33 sec.	1min.	1min.30sec.
40	3 sec.	13 sec.	40sec.	1min.
80	1 sec.	7 sec.	7sec.	18sec.

Thickness of samples	2mm		4mm	
Temperature (degree C)	Breaking time	Absolute dissolve time	Breaking time	Absolute dissolve time
5	3hr. 46min.	5hr. 18min.	Over 20hr.	Over 20hr.
20	44min. 10sec.	1hr. 40min.	5hr. 35min.	8hr. 10min.
40	31min	52min.	3hr. 55min.	4hr. 38min.
80	7min. 10sec.	12min. 36sec.	1hr. 10min.	1hr. 22min.

Supplementary Table 2 **Dissolving time for our fibers with different lengths under 50 °C.**

Length	Dissolving time (50 °C)
1 cm	20 min
5 cm	1 hr 15 min
10 cm	6 hr 30 min
15 cm	12 hr
20 cm	>18 hr

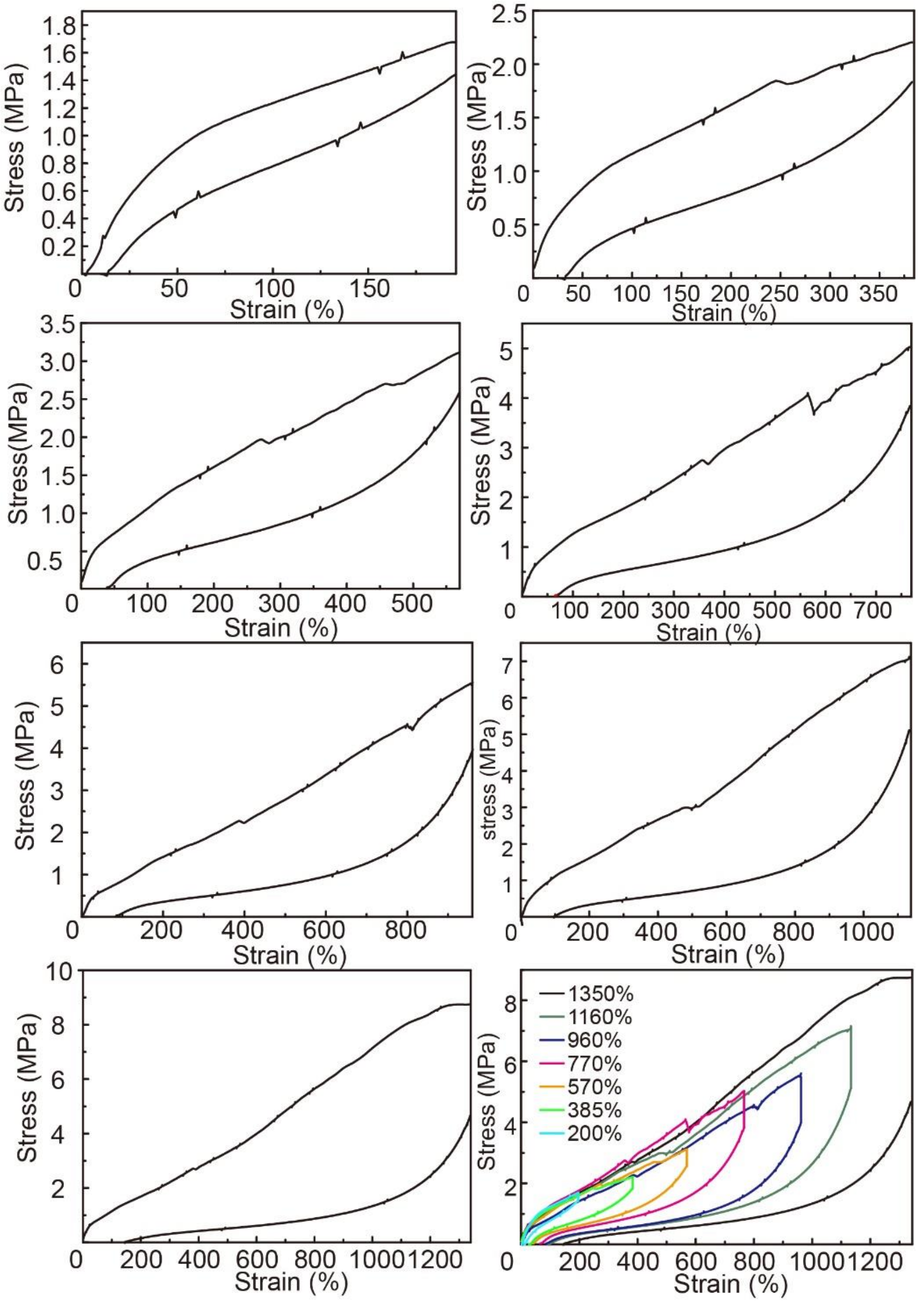

Supplementary Fig. 3 **Strain-stress cycle curves to test the resilience with stretching speed of 2 mm/s.** The resilience is a property of elastomers closely related to the block ratios, molecular weights, and molecular chain structures of polymers. Supplementary Fig. 3 shows the resilience tests through a strain-stress cycle curve of our fibers.

As observed from the strain-stress cycle curves, there is a small length difference could not be fully recovered. However, after being released from the clamps of the test setup, some of them could be fully restored to the original state under a moderate stretch. Supplementary Table 3 summarizes the length change of SEBS fibers after releasing with different recovering times. The stretchable SEBS fibers can fully recover to the original length within a short time when under moderate stretches (<600%), and take longer time to recover or may not fully restore under larger stretch conditions.

Supplementary Table 3 **Length change of SEBS fibers after releasing.**

Original 10 mm	Length start recovering	Length end recovering	Recovering time
200%	10 mm	10 mm	0
385%	10 mm	10 mm	0
570%	10 mm	10 mm	0
770%	11.5 mm	11 mm	7 min
960%	12 mm	11 mm	30 min
1160%	13 mm	12 mm	40 min
1350%	13 mm	12 mm	40 min

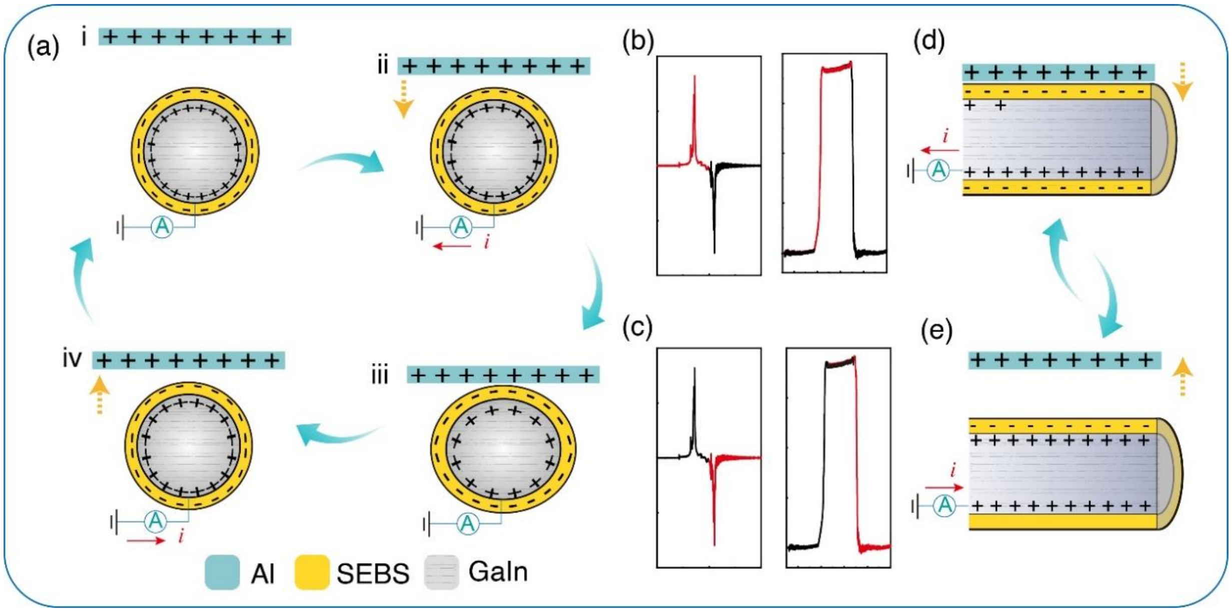

Supplementary Fig. 4 **Detailed schematic of the full working cycle mechanism of TENG fiber.**

Discussion 1: surface charge/area *v.s.* fiber length/ strains.

When a fiber is stretched, the volume remains the same, and the surface change trend can therefore be calculated. As surface charge is only related to the outer surface of the fiber, for simplicity, here we suppose the fiber is in solid state. As indicated in Figure S5, in the stretching process, the original length *L* is elongated to *L'*, the cross section area *A* and radius *R* are reduced to *A'* and *R'*, while volume *V* keeps the same, so we can derive how the outer surface *S* change with *L*.

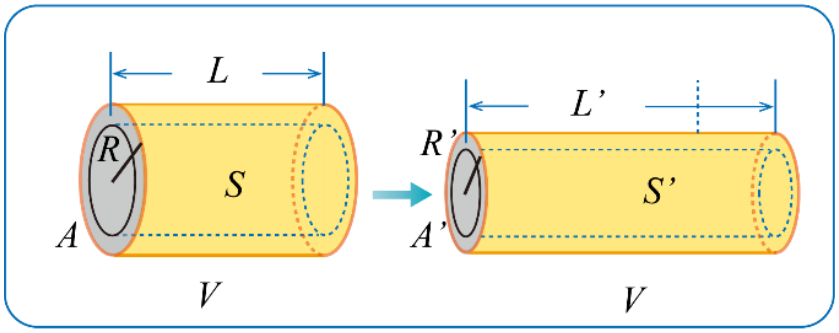

Supplementary Fig. 5 **Schematic of the fiber surface area change when being stretched.**

$$V = A \times L$$

$$A = \frac{V}{L}$$

$$\pi R^2 = \frac{V}{L}$$

$$R = \sqrt{\frac{V}{\pi L}}$$

$$S = 2\pi R \times L$$

$$= 2\pi\sqrt{\frac{V}{\pi L}} \times L$$

$$= 2\sqrt{\pi V L}$$

$$Q \propto S = 2\sqrt{\pi V L}$$

$$\Rightarrow$$

$$Q \propto S \propto \sqrt{L}$$

So, under an ideal condition, the surface charges and surface area are in a radicand relation with the fiber length. Thus, when fibers are stretched, the surface areas increase.

Other factors influencing the electrical output signals include fiber thickness, contacting efficiency, surface roughness, air humidity, environmental signals, etc. Among them, fiber thickness is the dominant factor when considering the changes with the stretch. So, we mainly discuss how the thickness *d* influence the surface charges. We define *r* is the inner radius, *R* is the outer radius, while V_1 and V_2 are the volume of fiber and core, respectively.

$$
\begin{aligned}
d &= R - r \\
&= \sqrt{\frac{V_1}{\pi L}} - \sqrt{\frac{V_2}{\pi L}} \\
&= \frac{\sqrt{V_1} - \sqrt{V_2}}{\sqrt{\pi L}}
\end{aligned}
$$

$$
d \propto \frac{1}{\sqrt{L}}
$$

So, the thickness decreases when the fiber length increases.

According to reported study,[3] when C approaches infinity, the maximum charge density in TENG can be expressed as:

$$
\begin{aligned}
\sigma_{\max, C \to \infty} &= \left(\frac{AP\varepsilon_0 (d + \varepsilon_r)}{d\left(\ln\left(Px\right) + B\right)} \right)_{\min} \\
&= \left(\frac{AP\varepsilon_0}{\ln\left(Px\right) + B} + \frac{AP\varepsilon_0 \varepsilon_r}{d\left(\ln\left(Px\right) + B\right)} \right)_{\min}
\end{aligned}
$$

Obviously, when C approaches infinity, the maximum charge density of CE-TENG (charge-excitation TENG) is equal to common TENG, and increases with the decrease of film (fiber) thickness *d*.

According to these studies, when the thickness is below 5 μm, the output increases greatly with the decreasing thickness. When the thickness is in 5~25 μm range, the output increases but not greatly. When the thickness is above 25 μm, the output does not change obviously. Our fiber thickness is about 100~150 μm thick, thus the thickness change has negligible influence on the electrical output.

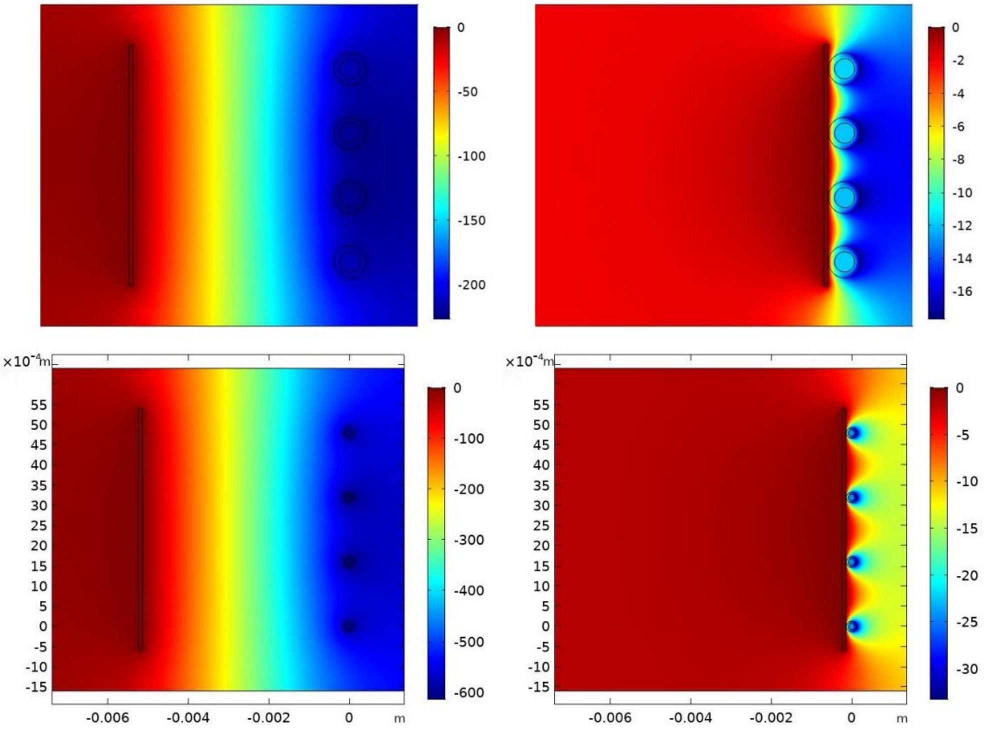

Supplementary Fig. 6 **Simulation results of 4 fiber in 0% and 1000% strain conditions.** When being stretched, a larger electrical potential can be obtained.

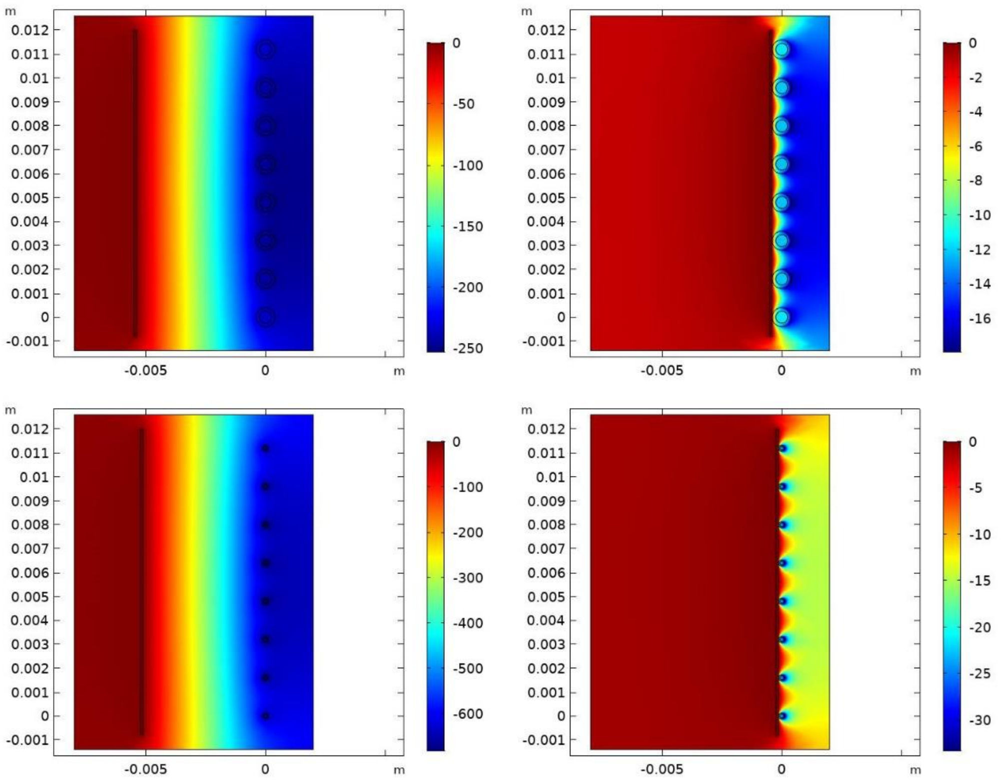

Supplementary Fig. 7 **Simulation results of 8 fibers under 0% and 1000% strain conditions.** Showing a larger output than fewer fiber number conditions, and when being stretched, a larger electrical potential is observed.

Discussion 2: signal errors introduced by random vibrations under high strains

As the increase of applied strains and the number of fibers, the random vibrations of fibers greatly increase (Figure S8a-b), leading to the change in current signal. The current signal was obtained through the rate at which charge flows through a given surface, so it was greatly influenced by this vibration. This random vibration cannot be avoided in the clamp-fiber-clamp system. Moreover, because of the superposition of randomness, it becomes more obvious when there are more fibers. The principle and unavoidability can be explained by **the fix-fix string vibration model** as simply schematized in Figure S8c, and the vibration frequency increases with string strains, thus resulting in the great change in Figure 3 in the manuscript. That's also the reason why we didn't take higher strain tests in Figure 3 though the fibers can still bear.

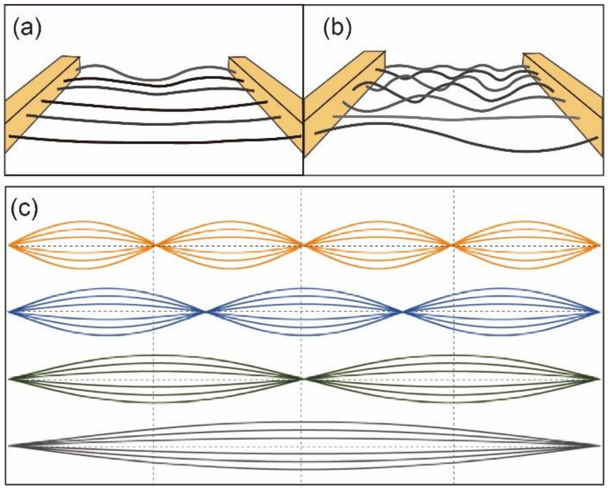

Supplementary Fig. 8. **Schematic to show the fiber vibrations.**

Now we use the basic fix-fix string vibration model to explain this phenomenon. If T is the tension in the string and m is its liner density, then the velocity (v) of the transverse wave along its length of a stretched fiber is given by the expression,[4]

$$v = \sqrt{\frac{T}{m}}$$

Here, for simply understanding, we only consider the fundamental mode of string vibration: the two ends are nodes and there is an antinode exactly midway between the two ends. In this mode, the frequency of vibration is the lowest. The distance between the two consecutive nodes is $\lambda/2$, and the length of the string (fiber) is l, and the vibration frequency is n.

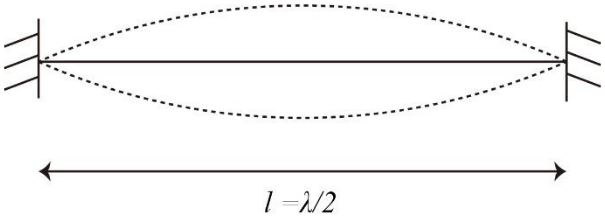

Supplementary Fig. 9 **Schematic of the basic fix-fix string vibration model.**

The frequency n of the fundamental mode of transverse vibration of a stretched string (fiber) is given by:

$$\lambda = 2l$$

$$while, v = n\lambda$$

$$v = n \cdot 2l$$

$$\text{now, } v = \sqrt{\frac{T}{m}}$$

$$n \cdot 2l = \sqrt{\frac{T}{m}}$$

$$n = \frac{1}{2l}\sqrt{\frac{T}{m}}$$

$$now,\ m = \frac{mass\ of\ fiber}{length\ of\ fiber}$$

$$= \frac{volume \times density}{length}$$

$$= \frac{area\ of\ cross\ section \times length \times density}{length}$$

$$= area\ of\ cross\ section \times density$$

$$= \pi r^2 \rho$$

By the definition of Young's Modulus, when L is the original length of the string (fiber) before stretching,

$$Y = \frac{stress}{strain} = \frac{F / A}{l / L}$$

$$T = F = tension\ in\ the\ fiber$$

$$= \frac{YAl}{L}$$

$$v = n \cdot 2l$$

$$\Rightarrow$$

$$n = \frac{1}{2l}\sqrt{\frac{\frac{YAl}{L}}{\pi r^2 \rho}} = \sqrt{\frac{Y\pi r^2 l}{4L^3 \pi r^2 \rho}}$$

$$n = \frac{1}{2L}\sqrt{\frac{Yl}{L\rho}}$$

Thus, for a stretchable fiber, the vibration frequency increases with its strain, which means that fibers under a higher strain can generate larger current signals due the random vibration than fibers under a lower strain. Such a random vibration induced current largely contribute the vibration of total current signals.

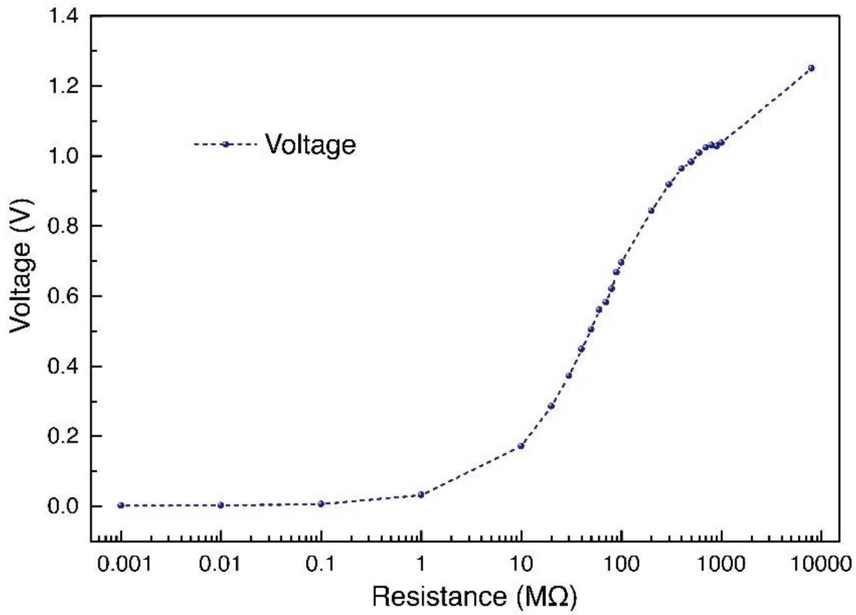

Supplementary Fig. 10 **Load voltages measured with different out loads.**

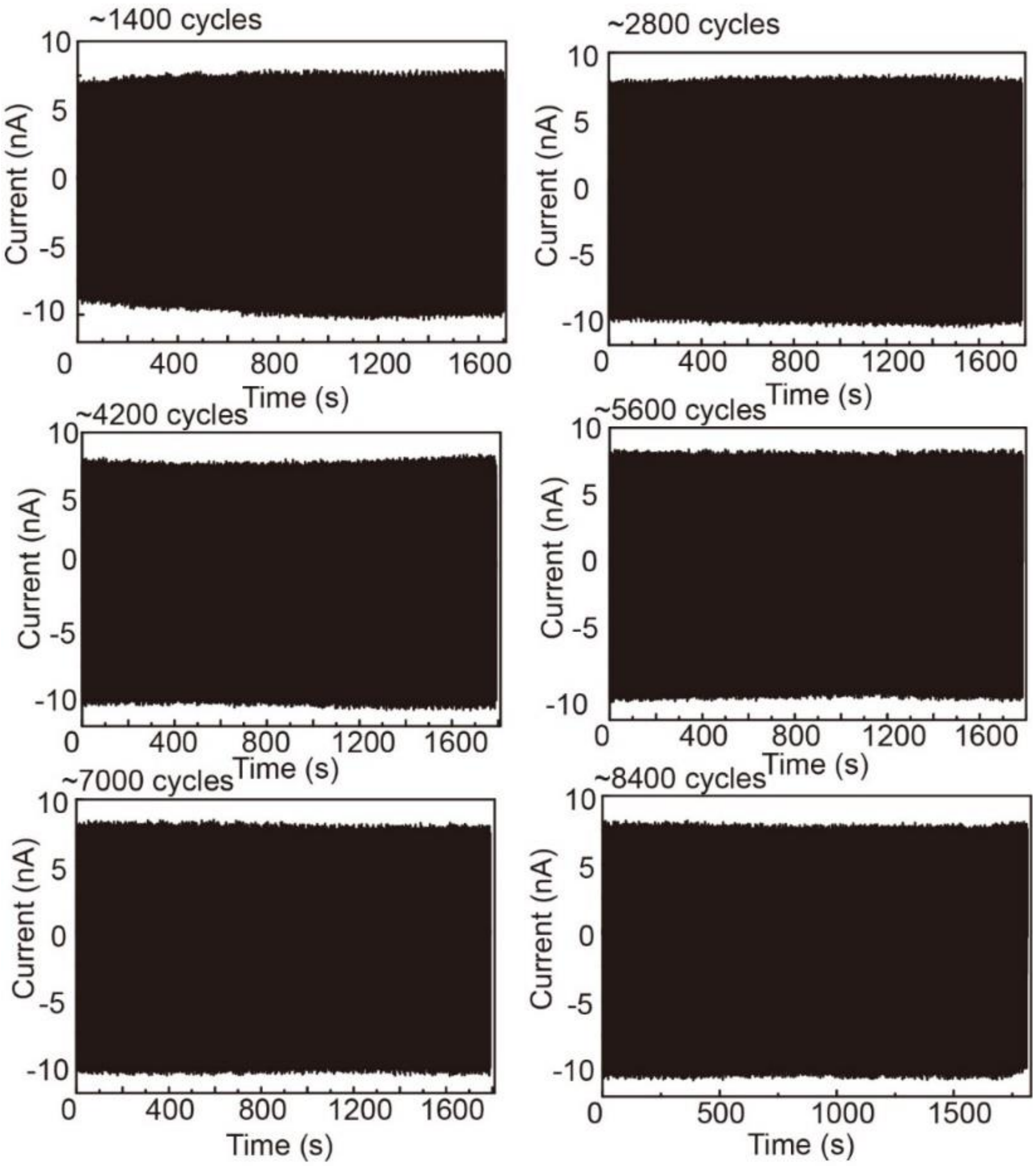

Supplementary Fig. 11 **Full current output with about 8400 contact-separate cycles to show its durability.**

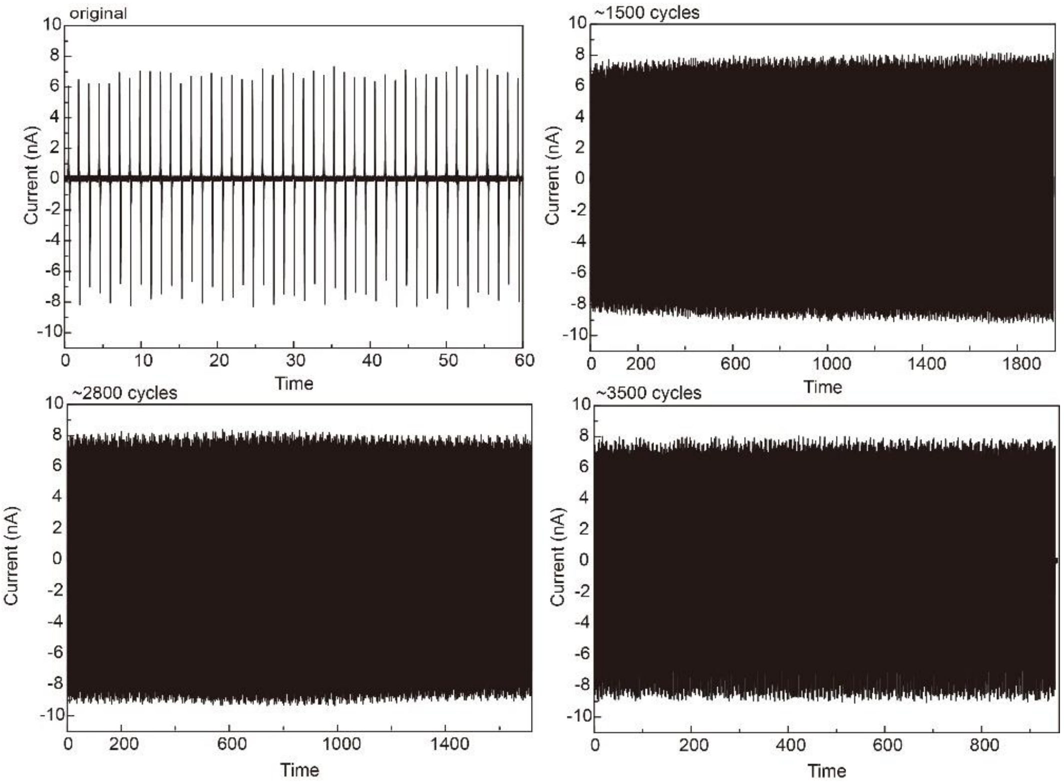

Supplementary Fig. 12 **Current output with ~3500 contact-separation cycles with 200% strain under 0.3 m/s speed to show the durability of the super-elastic conductive fibers.**

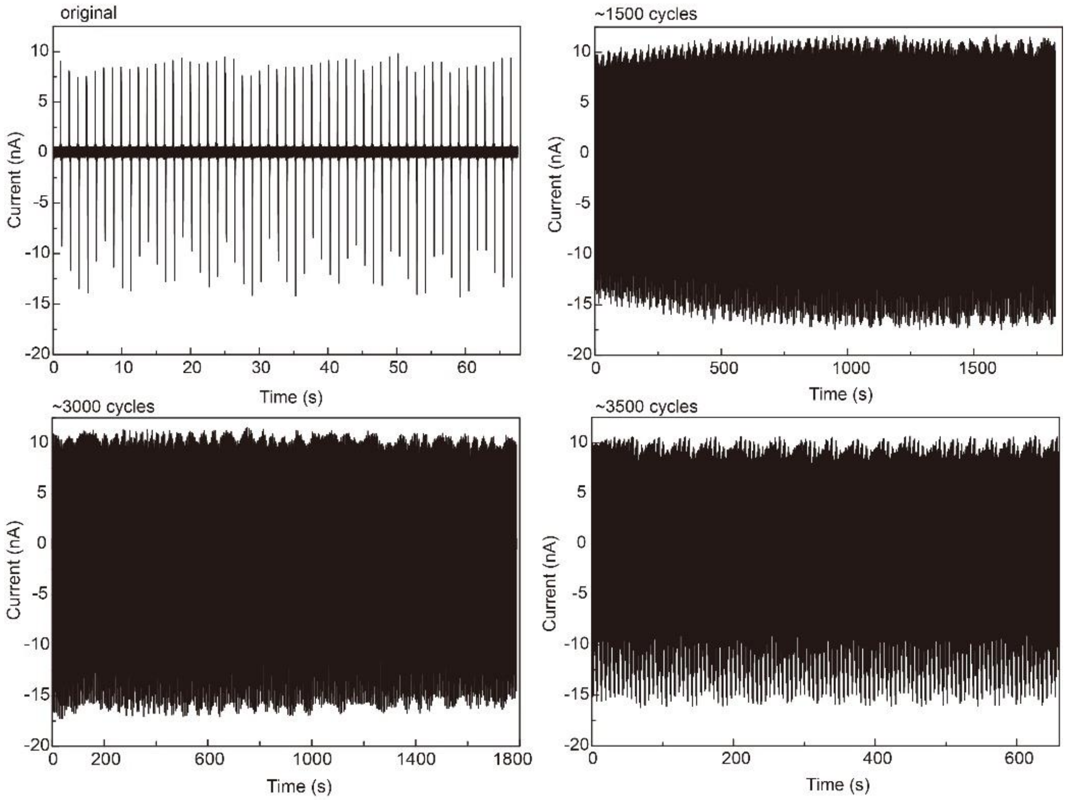

Supplementary Fig. 13 **Current output with ~3500 contact-separation cycles with 1000% strain under 0.3 m/s speed to show the durability of the super-elastic conductive fibers.**

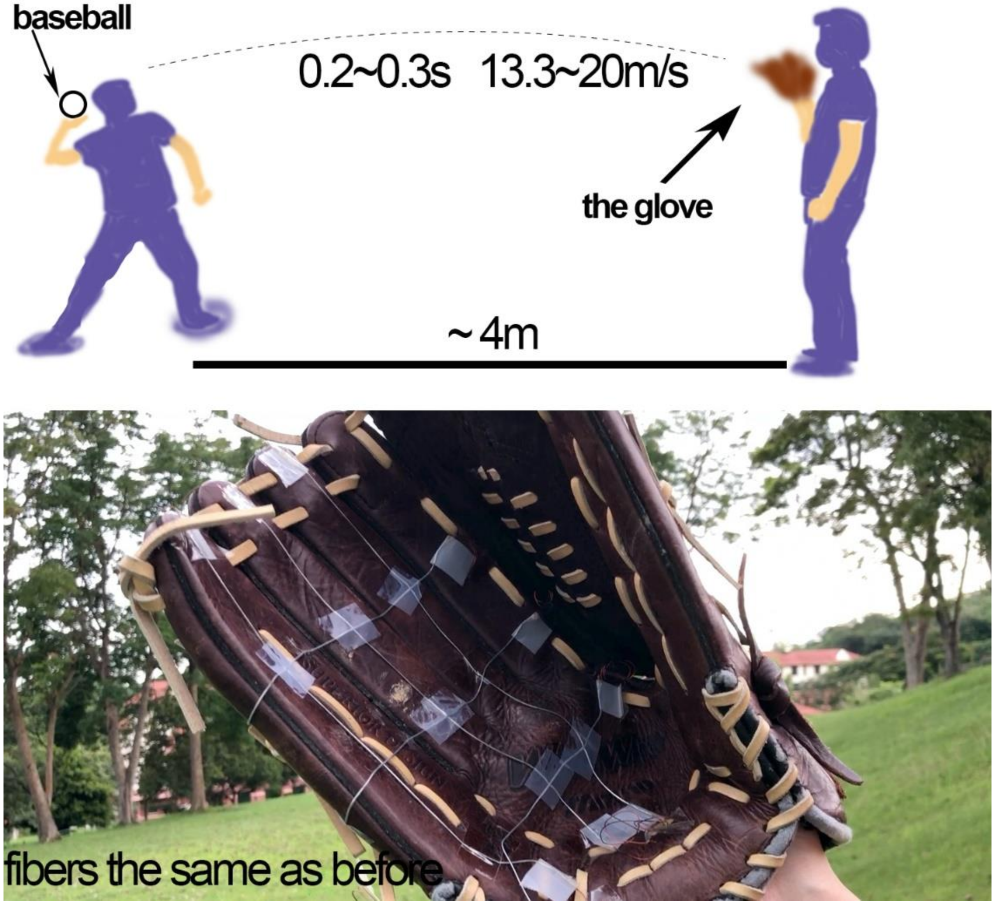

Supplementary Fig. 14 **Demonstration of feasibility under real baseball pitching and catching.**

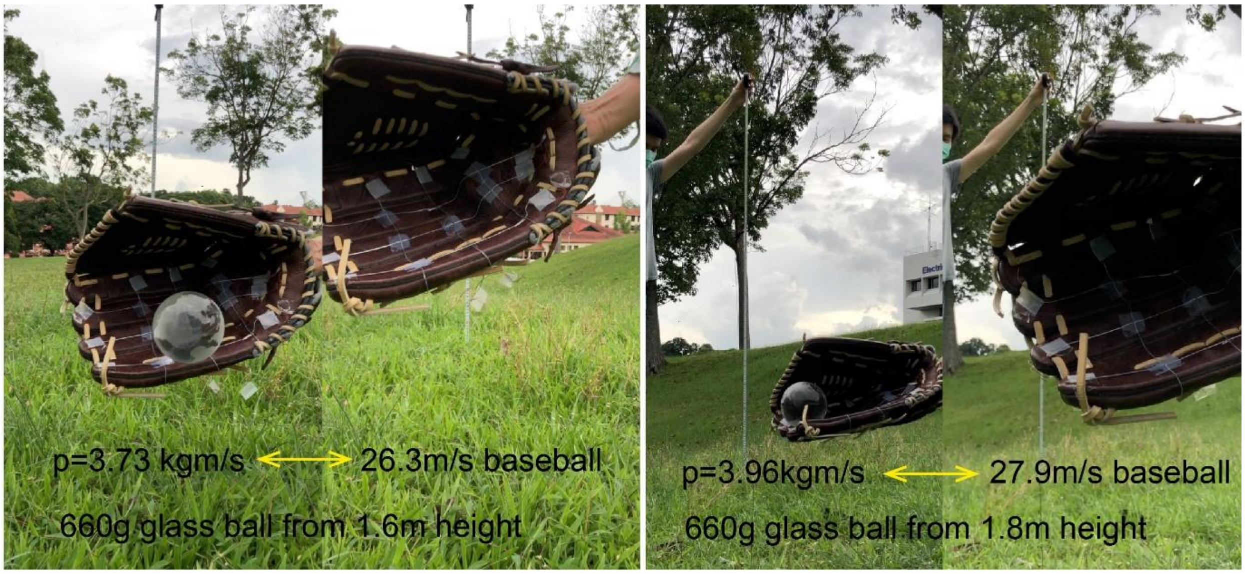

Supplementary Fig. 15 **Demonstrations of 660 g glass ball free falling from 1.6 m and 1.8 m heights, respectively.**

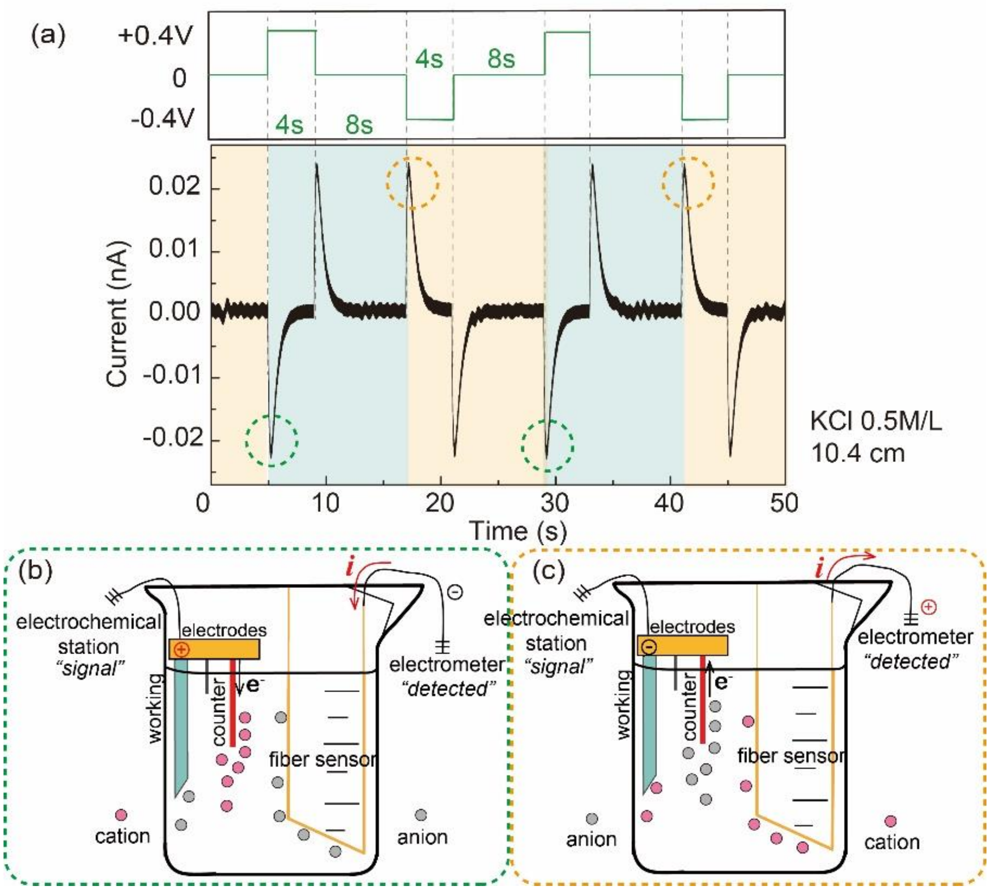

Supplementary Fig. 16 **Detailed schematic to explain the detecting processes.**

When "+0.4 V" is applied, the electrochemical station gives out electrons e^- with negative charges through the counter electrode (Pt wire). So, cations in the solution move from other locations toward the counter electrode, and anions distribute more at other locations in the solution. The equivalent effect is shown in Figure S16b. As there are negative anions outside the detecting fiber, positive charges move into fiber to reach balance. Thus, current direction is into the fiber from the electrometer, which appears as negative in the detecting signals (green dashed circles). When the applied source signal is fully applied, the solution system gradually reaches a balance state, and the detected peak recovers to 0. When the "+0.4 V" is off, an opposite process happens, and the detected signals become opposite.

When "-0.4 V" is applied, the electrons e^- with negative charges flow from the counter electrode (Pt wire) to the electrochemical station. So, anions in the solution move from other location toward the counter electrode, and cations distribute more at other locations in the solution. The equivalent effect is shown in Figure S16c. As there are positive cations outside the detecting fiber, negative charges move into fiber to reach balance. Thus, current direction is from the fiber into the electrometer, which appears as positive in the detecting signals (orange dashed circles). When the applied source signal is fully applied, the solution system gradually reaches a balance state, and the detected peak recovers to 0. When the "-0.4 V" is off, an opposite process happens, and the detected signals become opposite.

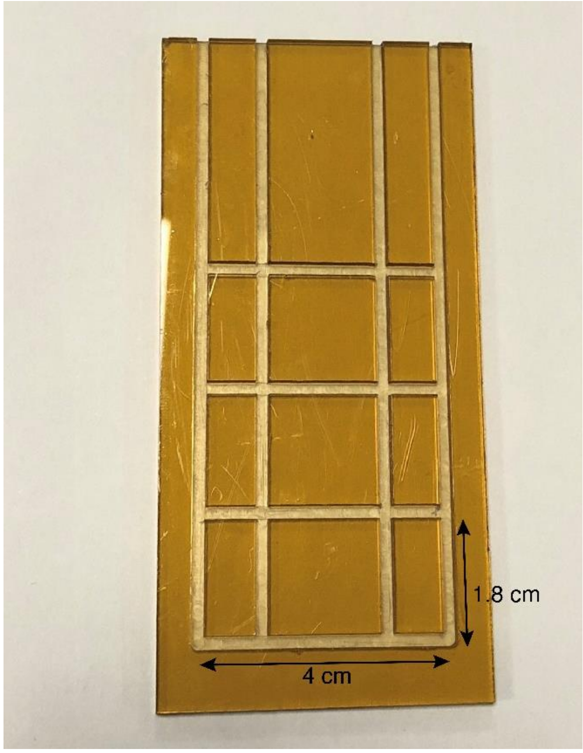

Supplementary Fig. 17 **Photo of the as fabricated fiber holder to modulate the immersed fiber length in solutions.** The soft fibers was placed in the grooves to be confined well, and by adjusting to a different groove, the immersed length is manipulated.

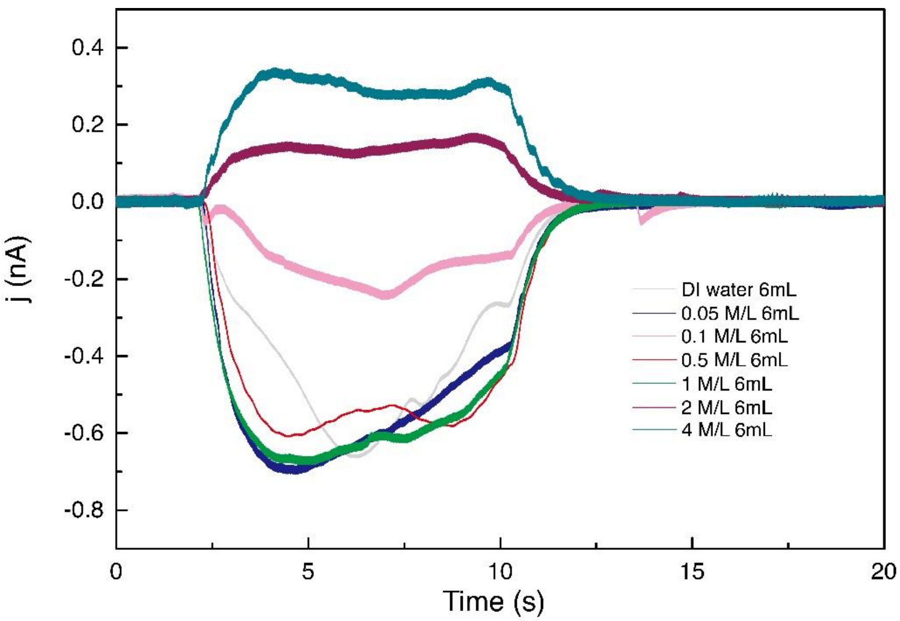

Supplementary Fig. 18 **Detection of *14* cm-long fiber immersed in 0.05 M/L NaCl solutions.** Use the injection pump to inject different concentration NaCl slolutions to induce ion concentration changes/variations into the solution at the same speed. Electrical signals induced by the concentration change be obviously observed.

References

1. Zhang Q, Shi C-Y, Qu D-H, Long Y-T, Feringa BL, Tian H. Exploring a naturally tailored small molecule for stretchable, self-healing, and adhesive supramolecular polymers. *Science advances* **4**, eaat8192 (2018).

2. Pingkarawat K, Bhat T, Craze D, Wang C, Varley RJ, Mouritz A. Healing of carbon fibre–epoxy composites using thermoplastic additives. *Polymer Chemistry* **4**, 5007-5015 (2013).

3. Liu Y, *et al.* Quantifying contact status and the air-breakdown model of charge-excitation triboelectric nanogenerators to maximize charge density. *Nat Commun* **11**, 1-8 (2020).

4. https://thefactfactor.com/facts/pure_science/physics/vibrations-of-string-harmonics-overtones/8410/.
