# Supplementary material for: Self-powered multifunctional sensing based on super-elastic fibers by soluble-core thermal drawing
Source: Nat Commun. 2021 Mar 3;12:1416. doi: 10.1038/s41467-021-21729-9 (PMC7930051; doi:10.1038/s41467-021-21729-9)
Supplement: Supplementary file 2 — Description of Additional Supplementary Files [file 41467_2021_21729_MOESM2_ESM.docx]

**Description of Additional Supplementary Files**

File Name: Supplementary Movie 1

Description: Up to 19 times stretching

File Name: Supplementary Movie 2

Description: Dumbbell freefalling.

File Name: Supplementary Movie 3

Description: Touched points on sphere
